# The effect of endurance training on levels of LINC complex proteins in skeletal muscle fibers of STZ-induced diabetic rats

**DOI:** 10.1038/s41598-020-65793-5

**Published:** 2020-05-26

**Authors:** Mehdi Bostani, Masoud Rahmati, Seyyed Ali Mard

**Affiliations:** 1 Department of Physical Education, Ahvaz Branch, Islamic Azad University, Ahvaz, Iran; 2 Department of Physical Education and Sport Sciences, Faculty of Literature and Human Sciences, Lorestan University, Khoramabad, Iran; 3 Alimentary Tract Research Center and Physiology Research Center, Department of Physiology, The School of Medicine, Jundishapur University of Medical Sciences, Ahvaz, Iran

**Keywords:** Physiology, Physiology, Health care, Diseases, Diseases

## Abstract

The changes of the linker of nucleoskeleton and cytoskeleton (LINC) complex have been studied in many muscular abnormality conditions; however, the effects of diabetes and physical activities on it have still remained to be defined. Therefore, the purpose of the this study was to evaluate the impacts of a six-week endurance training on the levels of SUN1 and Nesprin-1 proteins in Soleus and EDL muscles from diabetic wistar rats. A total number of 48 male Wistar rats (10 weeks, 200-250 gr) were randomly divided into healthy control (HC, N = 12), healthy trained (HT, N = 12), diabetic control (DC, N = 12), and diabetic trained (DT, N = 12) groups. Diabetes was also induced by a single intraperitoneally injection of streptozocin (45 mg/kg). The training groups ran a treadmill for five consecutive days within six weeks. The levels of the SUN1 and the Nesprin-1 proteins were further determined via ELISA method. The induction of diabetes had significantly decreased the levels of Nesprin-1 protein in the soleus and EDL muscles but it had no effects on the SUN1 in these muscles. As well, the findings revealed that six weeks of endurance training had significantly increased the levels of Nesprin-1 in DT and HT groups in the soleus as well as the EDL muscles; however, it had no impacts on the SUN1 in these muscles. The muscle fiber cross-sectional area (CSA) and myonuclei also decreased in diabetic control rats in both studied muscles. The training further augmented these parameters in both studied muscles in HT and DT groups. The present study provides new evidence that diabetes changes Nesprin-1 protein levels in skeletal muscle and endurance exercise training can modify it.

## Introduction

The nuclear envelope is a lipid bilayer membrane that separates the nuclear content from cytoplasm^1^. This membrane is composed of inner and outer parts, the physical connection within nuclear envelopes is mediated through linkers of nucleoskeleton and cytoskeleton, which are called LINC complexes ^2,3^. These LINC complexes are compounds of the SUN protein (sad1, and UNC-84) internally, and the KASH protein (klarsicht, ANC-1, and Syne/Nesprin-1, 2) externally^4^. The SUN proteins belong to the type 2 membrane proteins, which are generally found in all eukaryotic cells at the inner part of their nuclear envelope. Additionally, there are at least five different types of the SUN protein(SUN1-5) in mammalian genome^3^. The SUN1 with 182 amino acids and a 90 kD molecular weight is the largest isoform^5^. The SUN proteins (one of the two central component of the LINC complex) attaches the nucleus to the cytosol through cytoskeleton components (F-actin and intermediate filaments). These proteins are the main mediators for the mechanotransduction and physical pathways within cells and transmit mechanical stimuli to the nucleus. Accordingly, this fundamental role accounts for them as potential candidates for muscular abnormality conditions^6^. The KASH proteins are known as proteins of the outer nuclear membrane^7^. In this respect, one of the important components of the KASH protein is Nesprin1, which is also recognized as the synaptic nuclear envelope protein or Syne, ENAPTIN and NUANCE. These proteins belong to those found in the ONM. Thus far, six isoforms of the Nesprins have been documented as mammalian genome in which the Nesprin-1 is the largest isoform (1014 kD) ^8,9^. Nesprin-1 is found in many tissues, but its expression in skeletal muscles, cardiac, and vascular smooth muscle cells is higher than in other tissues^10^. Nesprins are also of utmost importance in maintaining the nuclear membrane, and cell processes such as muscle growth, cell replication, anchorage and nuclear positioning. Studies have shown anchorage and nuclear positioning are impaired in the skeletal muscles of rats with Nesprin-1 knockout ^8,10^. Nesprin-1 is also critical for the formation of the neuromuscular junction^10^. It has been further revealed that mutation in proteins of the nuclear membrane can lead to premature Progeria (HGPS), Emery Dreifuss Muscular Dystrophy (EDMD), Dunnigan-type familial partial lipodystrophy and cardiomyopathy. These diseases affect almost all systems in mammalian body, especially skeletal and cardiac muscle, lipid tissue, and peripheral nerves^11^.

The nuclear position within cells is determined by the connection of the LINC complex and the cytoskeleton ^12–14^. Proper position and docking of nuclei is critical for normal functions of skeletal muscles. Consequently, ablating or knocking out the Nesprin1 gene is associated with higher mortality, growth retardation, improper distribution and decreased number of nuclei in the tibialis anterior muscle, as well as a drop in exercise capacity in rats^10^. Improper nuclear positioning is also often associated with cell dysfunction, and it can have numerous clinical outcomes^12,15^. For example it has been demonstrated that there is a correlation between the occurrences of muscular diseases and improper nuclear positioning^12,16,17^.

In diabetes, high level of blood glucose triggers excessive stress and change the metabolic reactions which it in turn increases the level of reactive oxygen species^6^. This stress is more prominent in the involved tissues such as skeletal muscles which in turn damage the cellular structures^18^. Regarding to the critical role of Nesprin1 protein in positioning and anchoring of muscle nuclei^8,10^, and decreasing as well as dispositioning of nuclei in the diabetic-induced myopathy^19^, it seems that the levels of Nesprin1 in diabetic muscle fibers decreases too.

There are many investigations examining the effects of physical activities on various aspects of diabetes; however, to our knowledge, no studies have determined the impacts of diabetes on nuclear membrane proteins involved in nucleo-cytoskeleton. Therefore, the present study evaluated the changes in the LINC complex in diabetes for the first time. The other objective of this study was to investigate the effects of six-week endurance training intervention on the levels of the SUN1 and the Nesprin1 proteins in diabetic rats. Thus, we hypothesized that the diabetes and physical activity affect the proteins of LINC complex and then we analyzed the hypothesis.

## Materials and Methods

### Animals

A total number of 48 adult male Wistar rats were supplied from the Razi Institute (Karaj, Iran) and housed four per cage in an animal lab under standard conditions (12-hour light/dark cycle in a room at a temperature of 20–25 °C) with access to food and water ad libitum. All the institutional (as registered under the code LUNS.REC.1395.170 at Lorestan University of Medical Sciences) and animal research health guidelines were also observed. The animals were randomly divided into four groups: 1. Healthy control (HC, N = 12), 2. Healthy trained (HT, N = 12), 3. Diabetic control (DC, N = 12), and 4. Diabetic trained (DT, N = 12) followed by inducing diabetes in DT and DC groups.

### Diabetes induction

**Figure 1:**
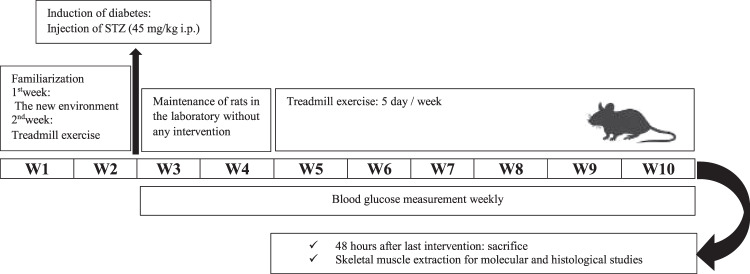
Study design.

For the purpose of acclimatization and reaching optimal weight (at least 250 gr), all of the rats were kept in an animal lab for two weeks prior to the experiments. Subsequently, following an overnight fast, diabetes was induced through a single intraperitoneal injection of streptozotocin (STZ) (45 mg/kg; Sigma, St. Louis, MO) solution (dissolved in 0.5 mol/L citrate buffer at pH 4.0). Two days later, diabetes was confirmed through measuring tail vein blood glucose level (>300 mg/dL) by Accu-Chek Compact Plus blood glucose meter (Roche Diagnostics K.K., Tokyo, Japan). In this regard, blood glucose levels were controlled throughout the study, once every week (Fig. 1).

### Exercise treadmill training episodes

The treadmill training protocol was developed based on previous protocols and consisted of six weeks of moderate-intensity endurance aerobic exercise on a level motor-driven treadmill (Model T510E, Diagnostic and Research, Taoyuan, Taiwan). The aerobic power of the animals was estimated based on the relationship between VO_2_max to speed and treadmill slope^20–22^. Within the first week, the speed and the duration of the treadmill running were 10 m/min and 10 min per day, respectively. This was then gradually increased until the fifth week a final training speed and duration of 18 m/min and 30 min per day, respectively. To stabilize the obtained adaptations, training speed and duration were kept constant during the sixth week (Table 1).

**Table 1:** Endurance Training Protocol.

	Sixth week	Fifth week	fourth week	third week	second week	first week
30	30	30	20	20	10	Training duration (minute)
18	18	15	15	10	10	Treadmill speed (m/min)

### Tissue extraction

Two days after the last exercise session, in the sixth week of training, the animals were anesthetized by inhalation of 2% halothane in a mixture of 20% O_2_ and 80% CO_2_. For the analysis of the SUN1 and the Nesprin-1 in the soleus and EDL, tissues of six rats were removed immediately and were stored at −80 °C until the analysis process was completed. Moreover, for immunohistochemistry (IHC) analyses, the soleus and EDL muscles of six rats were mounted on pieces of cork and fixed with tragacanth gum. Muscles were frozen in isopentane cooled by liquid nitrogen and further stored at −80 °C.

### Immunostaining analysis

Mid-belly muscle sections were cut into 10μm thickness by a Leica CM 3000 cryostat at −23 °C (Leica Microsystems, Wetzlar, Germany) and then they were stored at −80 °C until immunostaining. Frozen muscle cryosection were dried and were encircled with a Dako pen. Slides were chemically permeabilized with PBS containing Triton 0.5% for 15 min and then washed three times with PBS. Slides were blocked with bovine serum albumin 2% (BSA) for 1 h at room temperature and then incubated overnight with a rabbit anti-laminin primary antibody (L9393 Sigma-Aldrich, St. Louis, MO, USA, dilution 1/200) at 4 °C in a moist chamber. Slides were washed three times with PBS and incubated with anti-rabbit IgG Cy3-labeled secondary antibody (111-165-008, Jackson Immunoresearch Labs, West Grove, PA, USA, dilution 1/200) at 37 °C for 45 min. For SUN1/Laminin/DAPI or Nesprin1/Laminin/DAPI IHC, cryosections were labeled with antibodies against SUN1 (bsm-54420R) or Nesprin1 (ab192234) and laminin (L9393) overnight at 4 °C and labeling using the second antibody was performed for 2 h at 37 °C. Secondary antibodies were coupled to FITC or Cy3 (Jackson Immunoresearch Inc). Hoechst staining (1:1000, B2261, Sigma-Aldrich) was used to visualize nuclei and sections were mounted using Fluoromount G medium. Finally, slides were stored at 4 °C until picture acquisition.

### Immunohistochemistry quantification

For IHC, images were captured at ×10 and ×20 magnification at room temperature using a Carl Zeiss AxioImager fluorescent microscope (Carl Zeiss, Jena, Germany). Whole muscle sections were obtained using the mosaic function in Image M1 Software. Muscle fiber cross-sectional area was analyzed using Open-CSAM software^23^. This reliable and highly sensitive muscle analysis software has been validated against manual human counts and is both accurate and reliable. Laminin stained muscles were also used to quantify myonuclear number per fiber. Nuclei that clearly resided within the laminin border of the muscle fiber were scored as myonuclei using Image J software. To determine SUN1 and Nesprin1 density (SUN1 cells/fiber or Nesprin1 cells/fiber), SUN1 and Nesprin1 were counted manually using the cell tracker in ImageJ software and expressed as a percentage of total SUN1 and Nesprin1. For confocal analysis, pictures were taken on a TCS SP5 X microscope (Leica Microsystems) at 20X magnification. For each condition of each experiment, at least 8-10 fields chosen randomly were counted. The number of labeled SUN1 or Nesprin1 was calculated using the cell tracker in ImageJ software and expressed as a percentage of total SUN1 or Nesprin1. Finally, SUN1 or Nesprin1 counts were normalized to fiber number. All manual counting was performed by a blinded, well-experienced technician.

### Further assay of SUN1 and Nesprin-1 proteins

To support our results, we further analyzed the expression of the SUN1 and the Nesprin-1 proteins with ELISA kits (Cusabio-Japan). At the first step, the soleus and the EDL muscles were homogenized (1:10 in PBS 10 mM, pH 7.4 at 4 °C) and then centrifuged (20,000 rpm for 45 min). After that, using Rat’s ELISA kits (limit of detection for SUN1; and Nesprin-1 were 0.06 ng and 0.105 ng; respectively), the levels of proteins were measured.

### Statistical Analysis

Statistical analyses were conducted using SPSS Statistics software (Version 21, SPSS Inc., Chicago, IL, USA). Normality and homogeneity of the data were also assessed via Shapiro-Wilk test and Levene’s test, respectively. One-way analysis of variance (ANOVA) followed by Bonferroni post-hoc test was further employed to compare the SUN1 and the Nesprin-1 levels of the soleus and the EDL muscles and then compare the number of muscle nuclei in the HC, HT, DC, and DT groups. To assess the correlation between end-glucose levels and the SUN1 and the Nesprin-1 proteins, Pearson correlation coefficient was used and the statistical significance level was set by p < 0.05. The data were then reported as mean±standard error of measurement (SEM) values.

### Compliance with ethical standards

Ethics approval This study performed according to health guidelines for animal research and approved by Animal Care Committee of the Lorestan University (under the code LUNS.REC.1395.170),, and followed all the principals of the NIH Guidelines for the Care and Use of Laboratory Animals (NIH publication, 1996).

## Results

### Blood glucose levels response

**Figure 2:**
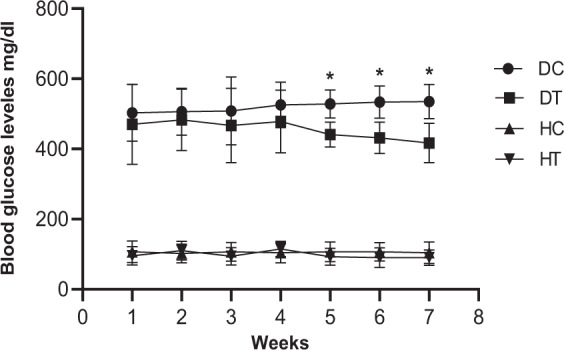
Blood glucose levels during the six weeks of endurance training. *P < 0.05 significant decrease in Blood glucose levels in diabetic trained (DT) versus the diabetic control (DC) group.

To evaluate the response of blood glucose to different treatments, we measured the blood glucose levels throughout the study. Figure 2 illustrates the average of blood glucose levels during the six-week endurance training intervention in the studied groups. As shown, blood glucose levels during the six weeks in the diabetic groups were always more than 300 mg/dL which was considered as the threshold for diabetes. In the diabetic control group, the blood glucose levels had been also rising since the injection of the STZ by the end of the sixth week; but in the diabetic trained group, a decreasing trend was observed which revealed a significant reduction started at the beginning of the fourth week of the study (p < 0.05).

### Effects of endurance training on levels of SUN1 and Nesprin1 proteins

**Figure 3:**
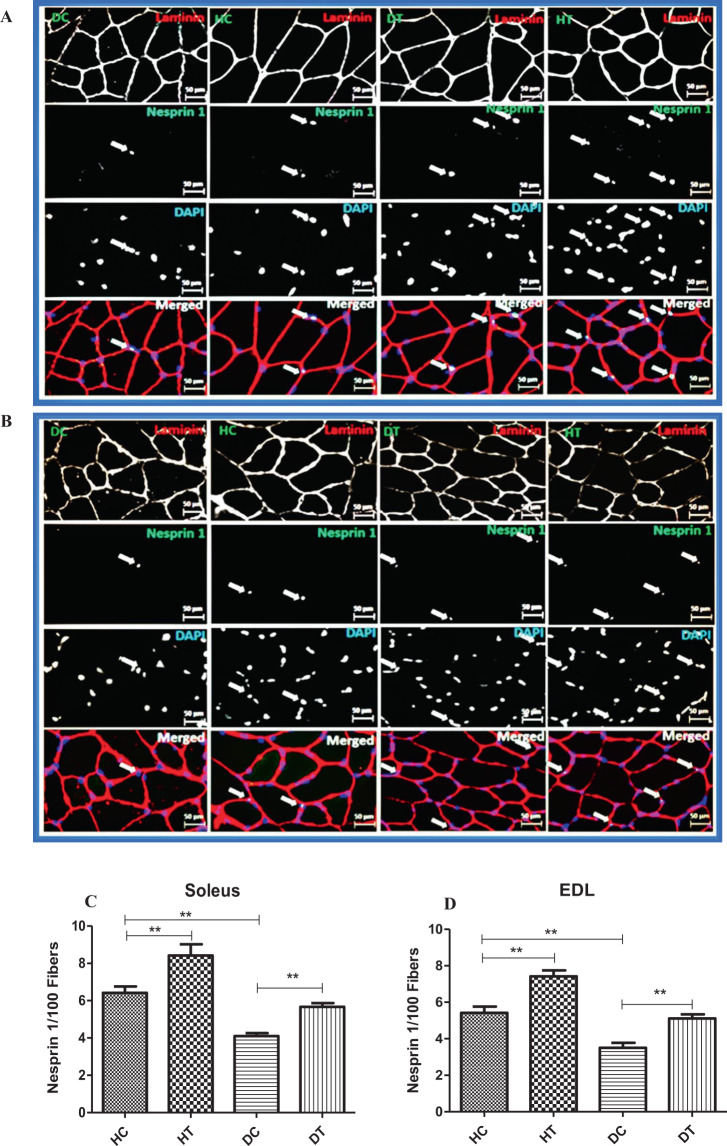
Immunostaining for Nesprin 1 in muscle sections. Sections of soleus (**A**) and EDL (**B**) muscles immunolabeled for Nesprin1 (green), Laminin (red), and Hoechst (blue). The number of Nesprin1^pos^ per fiber in Soleus (**C**) and EDL (**D**) muscles in the studied groups. Diabetes decreased the levels of the Nesprin-1 protein in the studied muscles while endurance training intervention for six weeks prevented this descending trend. **Significantly different, p ≤ 0.01. HC: Healthy control, HT: Healthy training, DC: Diabetic control, DT: Diabetic training.

**Figure 4:**
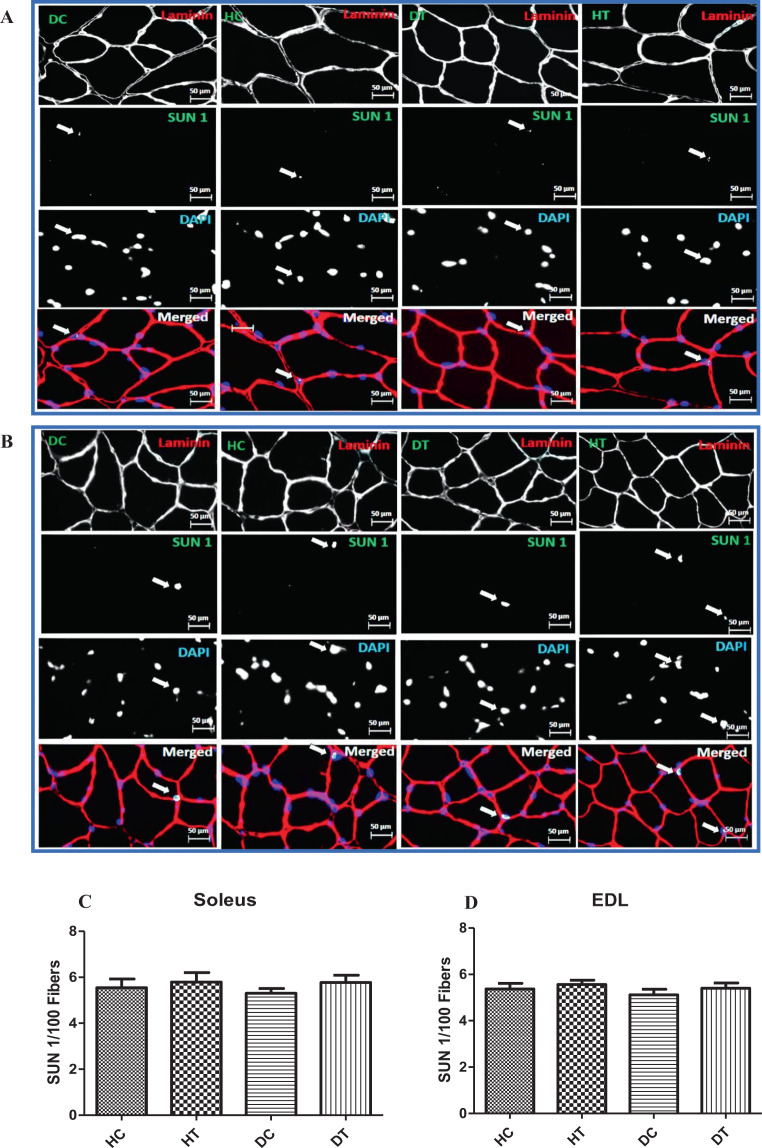
Immunostaining for SUN1 in muscle sections. Sections of soleus (**A**) and EDL (**B**) muscles immunolabeled for SUN1 (green), Laminin (red), and Hoechst (blue). The number of SUN1^pos^ per fiber in Soleus (**C**) and EDL (**D**) muscles in the studied groups. There was no significant difference between the groups in terms of protein expression of the SUN1 in the soleus and the EDL muscles of the male Wistar rats (p > 0.05). HC: Healthy control, HT: Healthy training, DC: Diabetic control, DT: Diabetic training.

**Figure 5:**
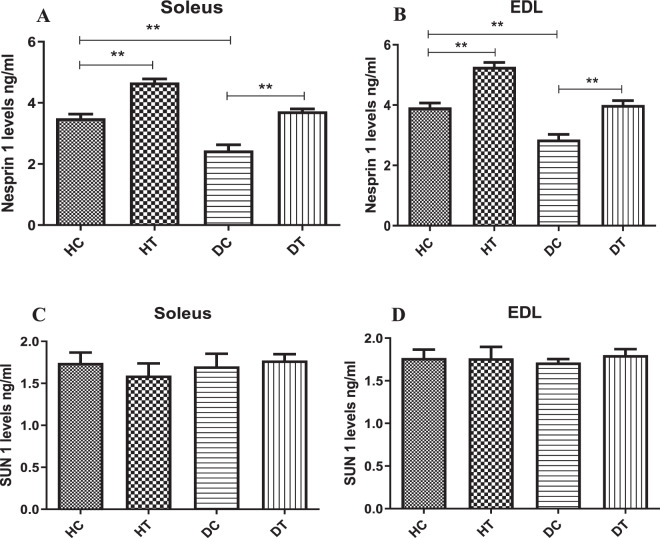
Assessment of Nesprin-1 and SUN1 proteins with ELISA method. Diabetes decreased the levels of the Nesprin-1 protein in soleus (**A**) and EDL (**B**) muscles while endurance training intervention for six weeks prevented this descending trend. There was no significant difference between the groups in terms of protein expression of the SUN1 in the soleus (**C**) and the EDL (**D**) muscles of the male Wistar rats (p > 0.05). **Significantly different, p ≤ 0.01. HC: Healthy control, HT: Healthy training, DC: Diabetic control, DT: Diabetic training.

To investigate the impacts of diabetes and exercise training on nuclear membrane proteins involved in nucleo-cytoskeleton, we next evaluate the levels of Nesprin-1 and SUN1. As displayed in Fig. 3, the levels of Nesprin-1 protein in the diabetic group were significantly lower than those of the control group in both the soleus (Fig. 3C) and the EDL (Fig. 3D) muscles (p < 0.01). In the HT group, undertaking six weeks of endurance training, the protein levels of the Nesprin-1 increased significantly. In animals in the DT group, a significant increase in the levels of the Nesprin-1 protein also occurred compared to the DC group. As demonstrated in Fig. 4, there was no significant difference in the expression of the SUN1 protein in the soleus (Fig. 4C) and the EDL (Fig. 4D) muscles between groups. That is, neither diabetes nor endurance training caused a significant change in the levels of the SUN1 in the soleus and the EDL muscles (p > 0.05). In addition, we found the same results for Nesprin-1 and SUN1 protein levels by ELISA method (Fig. 5). Diabetes decreased the levels of the Nesprin-1 protein in soleus (Fig. 5A) and EDL (Fig. 5B) muscles while endurance training intervention for six weeks prevented this descending trend. In addition, there was no significant difference between the groups in terms of protein expression of the SUN1 in the soleus (Fig. 5C) and the EDL (Fig. 5D) muscles (p > 0.05).

### Effects of endurance training on muscle size and myonuclear number

**Figure 6:**
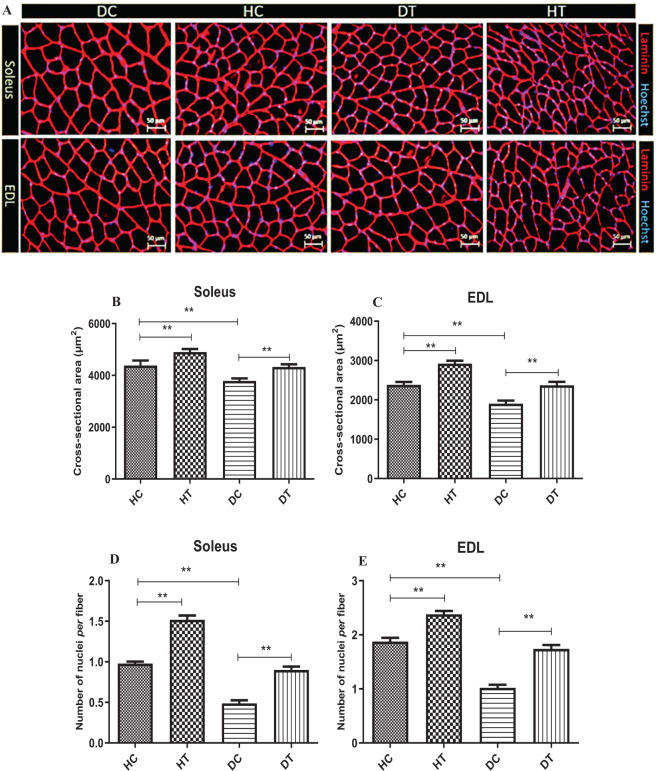
Muscle fiber cross-sectional area (CSA) in different groups. (**A**) Sections of soleus and EDL muscles immunolabeled for Laminin (red) and Hoechst (blue). Average muscle fiber CSA and myonuclei numbers decreased in soleus (**B** and **D**; respectively) and EDL (**C** and **E**; respectively) muscles of STZ-Induced diabetic rats. Compared to the healthy control group (HC), in the two groups that performed endurance training (HT and DT), CSA and myonuclei numbers significantly increased in both soleus and EDL muscles. **Significantly different, p ≤ 0.01. HC: Healthy control, HT: Healthy training, DC: Diabetic control, DT: Diabetic training.

An increase in myofiber size is a normal adaptation to increased workload. We assessed myofiber hypertrophy after 6 weeks endurance training in both the soleus and EDL muscles of diabetic and healthy rats. We next assessed the magnitude of myonuclear accretion after exercise training period. Average muscle fiber size (CSA) in response to diabetes and exercise is shown in Fig. 6. The CSA decreased significantly in the both soleus (Fig. 6B) and EDL (Fig. 6C) muscles of STZ-Induced diabetic rat compared to the healthy control group (HC) (p < 0.001 and p < 0.0001; respectively). In addition, compared to the healthy control group (HC), in the two groups that performed endurance training (HT and DT), CSA significantly increased (p < 0.001).

The number of myonuclei followed the decrease of CSA in STZ-Induced diabetic rats. The analysis of the data from muscle nuclei counts showed that the number of muscle nuclei in the diabetic group (DC) was significantly lower in both the soleus (Fig. 6D) and EDL (Fig. 6E) muscles from the healthy control group (HC) (p < 0.01). Moreover, in the two groups that performed endurance training (HT and DT), the number of muscle cell nuclei significantly increased (p < 0.01). This rising trend was observed in both the slow-twitch (soleus) and fast-twitch muscles (EDL) (Fig. 6).

### The relationship between end glucose levels and SUN1 and Nesprin1 proteins

**Figure 7:**
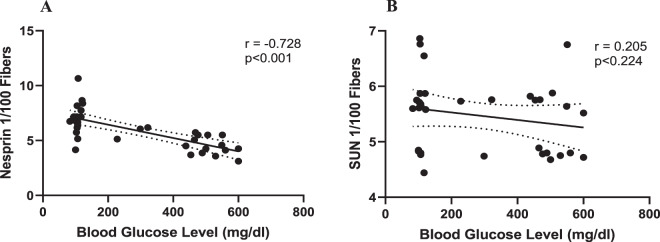
The results of the correlation between end blood glucose levels and Nesprin1 (**A**) and SUN1 (**B**) proteins. There was no correlation between blood glucose levels and the SUN1 protein. However, a strong and significant correlation was observed between end blood glucose levels and the Nesprin-1 protein.

Finally, we did a correlative analysis to examine the possible relationship between blood glucose levels and nuclear membrane proteins involved in nucleo-cytoskeleton in all groups (Fig. 7). There was no relationship between blood glucose levels and the levels of the SUN1 proteins (r = −0.205, Sig=0.224; Fig. 7A) in the studied muscles in diabetic groups at the end of the study. A strong and inverse correlation was found between final glucose levels and the expression levels of the Nesprin-1 protein (r = −0.728, Sig=0.001; Fig. 7B).

## Discussion

In this study, the results showed that the induction of diabetes had significantly decreased the levels of Nesprin-1 protein in the soleus and EDL muscles, but it had no effects on the SUN1 in these muscle. As well, the findings revealed that six weeks of endurance training had significantly increased the levels of Nesprin-1 in DT and HT groups in the soleus as well as the EDL muscles; however, it had no impacts on the SUN1 in these muscles. The muscle nuclei also decreased in diabetic control rats in both studied muscles. The training further augmented this parameter in both studied muscles in HT and DT groups. In this study, the expression of Nesprin1 protein was lower in diabetic versus healthy control rats. In addition, the present study showed that diabetes could significantly reduce the number of nuclei in muscle cells in both Soleus and EDL muscles. Since the Nesprin-1 plays an important role in the structure and the contraction of the muscles due to its linkage to the actin filament^24^, reducing the expression of this protein in diabetes could explain the structural weakness and the reduction of muscle contractility in diabetes, even though this issue had not been addressed in this study.

The Nesprin-1 is highly expressed in muscle tissues and has muscle-specific isoforms^25–27^. Some studies have suggested the structural organization of sarcomers and messengers between the extracellular compartment and nuclei for Nesprins^28,29^. In the present study, it was suggested that endurance training intervention could significantly increase the levels of the Nesprin-1 protein in the soleus and the EDL muscles.

On the other hand, due to the role of the Nesprin-1 protein in the positioning of the muscle nuclei, and according to the findings of this study which revealed the increasing number of the nuclei in muscle cells by endurance training, it was concluded that endurance activities could multiply the number of the nuclei in muscle cells and increases the Nesprin-1 protein levels. The distribution pattern and the distance of the nuclei from each other is also critical for facilitating the relationship between the nuclei to regulate and coordinate the expression of the protein and other natural actions of the cells^30^. In diabetes, the normal pattern was disrupted as shown in the present findings. However, in the group with endurance training, the pattern of nuclear distribution was reverted to near normal due to the increase in the levels of the Nesprin-1 protein. Therefore; sports activities, especially of endurance type, could be effective in maintaining the proper structure and the function of muscles in diabetes by changing the distribution and the number of muscle cell nuclei. These results were in agreement with the reports by Charifi *et al*.^31^, Fujimaki *et al*.^32^ and Snijders *et al*.^33^; attributed to increased diameter of muscle fiber and also increased the number of myonuclei per fiber following endurance training intervention. Therefore, it could be concluded that the physical activity could play an important role in increasing the expression of the Nesprin-1 protein in skeletal muscle.

The present study showed that physical activities could increase the levels of the Nesprin-1 protein in the soleous and the EDL muscles in diabetic rats. As discussed in the previous sections, the Nesprin-1 protein plays an important role in positioning of the muscle nuclei, as well as maintaining muscle structure and contractile functions. Likewise, considering the important role of the muscle nuclei in cell devision as well as preservation of DNA integrity^34^, and in the normal functioning of the muscles, such as protein synthesis, gene expression, intracellular transfer, cell division, migration, differentiation, fertility, and polarization^35,36^; it was concluded that physical activities could increase the levels of the Nesprin-1 by adding the number of nuclei of muscle cells and also their proper positioning in diabetic muscle fibers.

In this study, no changes in SUN1 protein were observed due to diabetes and endurance training. It seems that the inner membrane proteins of LINC complexes are less affected by diabetes or mechanical stimulus such as exercise. Perhaps the type of exercise and its intensity may not have been sufficient to affect this protein. thus, further research is needed in this field.

Physical activity is one of the major causes of physiological stress and impairment in blood glucose levels hemostasis, which can be different depending on the speed and the force of contraction of the muscles, the sources of the energy used, as well as changes in blood glucose especially in diabetics^37^. This study revealed that the glucose levels in diabetic rats had significntly decreased 4 weeks after physical training. It seemed that this descending trend was due to an increment of insulin sensivity secondary to physical training.

## Conclusion

The Nesprin-1 protein is one of the major components of the LINC complex which is located in the outer membrane of the nucleus. The main role of this protein is the proper posiotioning of the muscle nuclei. Furthermore, the proper placement of muscle cells for normal muscle function is essential. In the current study, diabetes reduced the levels of this protein in the soleus and the EDL muscles. The present findings showed that physical activity, as a non-pharmacological strategy, could increase the number of muscle cell nuclei, the levels of the Nesprin-1 protein and, muscle fiber size in diabetes.
